# Validation and reproducibility of a new iodine specific food frequency questionnaire for assessing iodine intake in Norwegian pregnant women

**DOI:** 10.1186/s12937-019-0489-4

**Published:** 2019-10-29

**Authors:** Synnøve Næss, Inger Aakre, Marian Kjellevold, Lisbeth Dahl, Ive Nerhus, Lisa Kolden Midtbø, Maria Wik Markhus

**Affiliations:** Institute of Marine Research (IMR), 5817 Bergen, Norway

**Keywords:** Iodine, FFQ, Validation, Pregnancy, Dietary assessment

## Abstract

**Background:**

Iodized salt is not mandatory in Norway, and the permitted level of iodine in table salt is low (5 μg/g). Thus, milk and dairy products, fish and eggs are the main dietary sources of iodine in Norway. Mild-to-moderate iodine deficiency in pregnant women has been described in several European countries, including Norway. There are few validated tools available to assess iodine intake in an efficient manner. The aim of the current study was to assess the validity and reproducibility of a new iodine-specific food frequency questionnaire (I-FFQ) in Norwegian pregnant women.

**Methods:**

An I-FFQ consisting of a total of 60 food items and the use of supplements was developed to assess iodine intake and was administrated to 137 pregnant women at gestational week 18–19. Reference methods were a structured 6-days iodine specific food diary, urinary iodine concentration (UIC) (pooled sample of spot UIC from six consecutive days), and thyroid function tests. Correlation analyses, Cohen’s weighted kappa, Bland-Altman plots, and linear regression analyses were used to assess validity. Reproducibility of the I-FFQ was assessed in a subgroup (*n* = 47) at gestational week 35–36.

**Results:**

There was a strong correlation between estimated iodine intake from the I-FFQ and food diary (*r* = 0.62, *P* < 0.001) and an acceptable correlation between the I-FFQ and UIC (*r* = 0.21, *P* = 0.018). There was no significant association between the I-FFQ and thyroid function tests. The I-FFQ estimated higher iodine intake compared to the food diary with a mean absolute difference 33 μg/day. The limits of agreement from the Bland-Altman plots were large, however few participants fell outside the limits of agreement (5.2–6.5%). There was no difference between the estimated iodine intake from the I-FFQ assessed at gestational week 18–19, and gestational week 35–36 (*P* = 0.866), and there was a strong correlation between the two time points (*r* = 0.63, *P* < 0.001).

**Conclusion:**

In summary, this study suggests that the I-FFQ can be used as a valid tool to estimate and rank iodine intake among Norwegian pregnant women. We further suggest that this I-FFQ may also be valid in other populations with similarly dietary patterns and where salt is not iodized.

**Trial registration:**

The study is registered in ClinicalTrials.gov (NCT02610959).

## Introduction

Iodine is an essential micronutrient which is crucial for the synthesis of the thyroid hormones triiodothyronine (T3) and thyroxine (T4) [1]. The thyroid hormones regulates a wide range of physiological functions in the body and are essential for normal growth, development and metabolic regulation [2]. Although great progress have been made towards eliminating iodine deficiency, it is still present in many European countries [3]. Furthermore, pregnant women are a particularly vulnerable group regarding iodine deficiency, as the fetus is dependent on supply of thyroid hormones and iodine from the mother [4]. Mild- to moderate iodine deficiency has been described in several studies of Norwegian pregnant women [5–8]. Iodized salt is not mandatory in Norway, and the permitted level of iodine in table salt is low (5 μg/g), therefore the main dietary sources of iodine in the Norwegian diet are milk and dairy products, fish and eggs [9, 10]. Since 2018, women of childbearing age in Norway with a low intake of milk and fish have been advised by the Norwegian Directorate of Health to take iodine containing supplements in order to secure sufficient iodine intake (supplement of 150 μg/day for pregnant and lactating women and supplement of 100 μg/day for women in fertile age) [11]. This recommendation was introduced after the current study was conducted.

Urinary iodine concentration (UIC) is the most common method for assessing iodine status [1]. However, as iodine excretion in urine varies considerable in individuals both between days, and within the same day, spot urine is not recommended to assess individual iodine status [12]. The day-to-day variation will neither be accounted for in one 24-h urine sample [12, 13]. Other biomarkers such as thyroid stimulating hormone (TSH) and thyroglobulin may also be used to assess iodine status, but all current methods poses limitations [1, 12]. Dietary assessment methods may be used to estimate iodine intake, to identify the major dietary iodine sources, and evaluate the iodine status through dietary recommendations [12]. However, all dietary assessment methods poses several challenges, as many tools are time consuming for the participants and rely on memory and accurate frequency, or portion estimations [13]. Further, dietary methods are highly dependent on accurate and reliable food composition data [12]. This may pose an extra challenge for assessment of iodine intake, since iodine content of food may vary considerably, e.g. between similar products [14] or between the same species of fish [15]. Food frequency questionnaires (FFQs) are one of the most common dietary methods used to measure dietary intake in nutrition research [16]. FFQs are particularly useful for assessing food items which are not frequently consumed, or foods consumed by less than 50% of the population that contain high levels of nutrients [17]. FFQs can further be used to assess habitual iodine intake [12], however all FFQs developed should be validated for the specific nutrient and population [18].

The Mommy’s Food study is a randomized controlled trial (RCT), designed to explore whether cod intake during pregnancy could affect iodine status and subsequently infant development [19]. An iodine-specific FFQ (I-FFQ) was developed for the RCT in order to assess the habitual iodine intake from iodine-rich food groups (milk and dairy products, seafood, eggs) and supplements in pregnant Norwegian women [19]. The I-FFQ was based on a short validated seafood FFQ for pregnant and post-partum women [20]. A full FFQ has previously been used and validated to assess iodine intake in pregnant women in Norway [21, 22]. However, a full FFQ may be very comprehensive and time consuming for the participants. An I-FFQ has the advantage to measure long-term dietary iodine intake in a simple, cost- and time-efficient manner, in a large sample size that is geographically widespread [16]. It can also be useful in identifying the most important sources of iodine in the diet [23]. Continuously monitoring of iodine intake in a population is important, especially in groups where deficiency already has been ascertained, such as pregnant women in Norway and in Europe.

As iodine deficiency is re-emerging in Norway, an I-FFQ to assess and evaluate iodine intake may be a useful tool. The aim of the present paper is to assess the validity and reproducibility of a new I-FFQ against the following: (1), a 6-days iodine specific food diary (2), urinary iodine concentration (UIC) (3), thyroid function tests (thyroid stimulating hormone (TSH), free T3 (fT3), free T4 (fT4)).

## Methods

### Study design and subjects

This paper used data from the Mommy’s Food study, a two-armed RCT in Norwegian pregnant women. The primary and secondary outcomes of the study were to investigate if an increased intake of cod during pregnancy has an impact on i) maternal iodine status and ii) infant development. The overall study design, including enrolment, randomization, study procedure and ethics are further described in detail by Markhus et al. [19]. An overview of methods used in the validation of this I-FFQ is shown in Figure S1, listed in Additional file 1. This current paper used data from baseline (pre-intervention) at gestational week 18–19 for assessing validity of the I-FFQ. A total of 137 pregnant women from Bergen, Norway were enrolled in the study. The participants were recruited between January 2016 to February 2017. Data from the I-FFQ were available from 124 participants, food diary from 134 participants and UIC from 134 participants. The sample size was considered adequate in according to validate an FFQ in a population group, where of at least 50–100 subjects is recommended [18]. To assess reproducibility of the I-FFQ, data from the control group post-intervention (gestational week 35–36) were used. Due to the design of the study (RCT) we were only able to use the control group (*n* = 47) for assessing reproducibility as the intervention group was intended to increase their iodine intake. Thus, including the intervention group would not be suitable for assessing reproducibility.

The study was conducted and performed according to the Declaration of Helsinki. The study was approved by the Regional Committees for Medical and Health Research Ethics West (REK 2015/879) and is registered in ClinicalTrials.gov (NCT02610959).

### Data collection methods

The participants received an electronic online questionnaire in gestational week 18–19 and gestational week 35–36. The questionnaire included questions of baseline characteristics such as age, gestational age, education level, nicotine use, pre-pregnancy weight, current weight and height. Pre-pregnancy body mass index (BMI) was calculated as pre-pregnancy weight in kilograms (kg) divided by the square of the height in meters (kg/m^2^).

#### I-FFQ

A semi-quantitative I-FFQ were included in the online questionnaire to acquire information about the participants’ habitual diet and supplement use with focus on iodine rich food groups. In the I-FFQ completed in gestational week 18–19, the participants were asked to report an estimate of their diet since they became pregnant. In the I-FFQ completed in gestational week 35–36, the participants were asked to report an estimate of their diet the last 16 weeks (since the last time they completed the I-FFQ).

The number of food items listed in the I-FFQ and the number of responses of frequencies for each food items are listed in Additional file 1: Table S1. The I-FFQ included 21 food items regarding frequency of seafood intake as dinner and warm lunch, and 14 food items regarding frequency of seafood intake as spread. All questions included type of seafood species or products consumed. The frequency intervals ranged from *“never”* to *“three times a week or more”* (five frequency alternatives in total). Further, there were 24 food items regarding intake of milk and dairy products (including mixed foods with milk such as pancakes, waffles and porridge) with frequency intervals ranging from *“never”* to *“three to four times per day”* (seven frequency alternatives in total). All food items of seafood, milk and dairy products had follow-up questions concerning portion sizes per meal, ranging from *“half a portion or less”* to *“three portions”* (five portion alternatives in total)*.* There was in addition one food item regarding weekly intake of eggs ranging from *“less than one egg per week”* to *“eight or more per week”* (six alternatives in total). Thus, a total of 60 food items, regarding iodine rich foods (fish and seafood, milk and dairy products and eggs), were included when calculating total iodine intake from the diet from the I-FFQ. In addition, the I-FFQ also included questions regarding dietary supplements including type, brand and intake frequency.

Data from the I-FFQ were converted to numerical continuous data through calculation of indexes in accordance to the methodology described in Markhus et al. [20]. For seafood intake, when frequency of consumption was given as a range (e.g. 1–2 times per week), the lowest frequency in each range was used (*here*: 1 time per week). This was due to recall is prone to overestimate low intakes when asked about several detailed food items separately [20]. For milk, dairy products, eggs and dietary supplements, when frequency consumption was given as a range (e.g. 1–2 times per day), the mean frequency consumption was used (*here*: 1.5 times per day) as these food categories consisted of fewer detailed questions. The calculated frequency indexes of each question were further multiplied by the reported portion sizes of intake to estimate weekly intake.

#### Food diary

A structured manual 6-days food diary was handed out, and instructions from the researcher were given to the participants at the first visit in gestational week 18. The food diary was filled out on six consecutive days between gestational week 18–19 (exact same days as the spot urinary samples). The food diary was developed specifically for this study with the purpose to estimate iodine intake during pregnancy. The number of food items listed in the food diary is given in Additional file 1: Table S1**.** The food diary included eight questions regarding seafood intake (as lunch/dinner and as spread), 19 questions regarding milk and dairy products, including foods made with milk, such as pancakes, waffles and other mixed dished with milk, and one question about intake of eggs. Thus, a total of 28 food items, regarding iodine rich foods (fish and seafood, milk and dairy products and eggs), were included when calculating total iodine intake from the diet from the food diary. The food diary also included questions regarding use of dietary supplements including type, brand and intake frequency. Each question included quantitative response alternatives (portions, glasses or cups, slices etc.) and the participants filled out their respective intakes from each day (e.g. *4* glasses of milk).

#### Estimation of iodine intake from the I-FFQ and food diary

In order to calculate consumption of the different food items from the I-FFQ and the food diary in grams per week, intake in portions per week were multiplied by estimated portion sizes in gram as defined in the report “Weights, measures and portion sizes for foods” from the Norwegian Food Safety Authority, University of Oslo and the Norwegian Directorate of Health [24]. These portion sizes were also defined in the I-FFQ and the food diary. To calculate the mean daily iodine intake from the I-FFQ and food diary, intake of the different food groups in gram per day was multiplied by the average iodine content of the specific food and further summarized. The iodine content of the specific food groups were retrieved from Nerhus et al. 2018 [15], the database *Seafood data* from the Institute of Marine Research (IMR) [25] and the Norwegian Food Composition Table [26]. The most relevant and recent analytical value of iodine was used. Information of iodine content of the specific food groups are specified in Additional file 2: Table S2 and S3. Regarding dietary supplements, the specific iodine content of each supplement was retrieved from the manufacturers, and the mean daily iodine intake from supplements were calculated from both the I-FFQ and the food diary. Total estimated iodine intake per day from both the I-FFQ and the food diary were further calculated by summarizing the estimated iodine from foods and estimated iodine intake from supplements.

#### Urinary iodine concentration and thyroid function tests

The participants collected one spot urine samples on six consecutive days between gestational week 18–19 (same days as the structured 6-days food diary). The participants were instructed to collect the spot urine sample between 4 PM and midnight. The participants stored the urine samples in their home freezer until the next visit in gestational week 19. The urine samples were then transferred to cryo tubes and stored at minus 20 °C pending analysis by inductively coupled plasma mass spectrometry (ICP-MS), at the Institute of Marine Research, Norway. Equally amounts of urine from the six spot urine samples were homogenized into one composite sample of 1 ml before determination of iodine concentration. Description of the analytical method to determine UIC (μg/L) has previously been described by others [7, 27]. Estimated iodine intake from UIC was calculated by the following formula: UIC (μg/L) × 0.0235 x body weight (kg) [28], using current pregnancy weight in kg as body weight (self-reported body weight from gestational week 18–19).

Blood samples were drawn at gestational week 18. Venous blood samples for serum preparation were collected in BD Vacutainer® SST™ vials II *Advanced* and set to coagulate for minimum 30 min before centrifuging (1000–3000 G, room temperature, 10 min) within 60 min after extraction. Post separation, serum samples were stored at minus 80 °C pending analysis at Fürst Medical Laboratories in Bergen, Norway. The serum samples were stored for maximum three months before analysis. TSH, fT4 and fT3 were analysed in serum using magnetic separation and detection by chemiluminescence, labelled with acridinium ester, on an Advia Centaur XPT Immunoassay system (Siemens Healthcare diagnostics Inc., Tarrytown, USA). For all blood constitutes the CV was < 6%.

### Statistics

Statistical analyses were performed using Statistical Package for Social Sciences version 25, IBM Corporation (IBM Corp. Released 2017. IBM SPSS Statistics for Windows, Version 25.0. Armonk, NY: IBM Corp). Two-sided statistical tests were performed. *P*-values < 0.05 were considered statistically significant. Variables were tested for normality by using the Kolmogorov-Smirnov test and by visual inspection of Q-Q plots and histograms. Descriptive results are reported as frequency (%) for categorical variables. For continuous variables mean (SD), median (p25-p75) or p5-p95 are reported as appropriate. Difference between estimated iodine intake from the I-FFQ, food diary and UIC were assessed using Wilcoxon signed-rank test. Correlation between the methods were assessed using Spearman’s rank order correlation coefficient (Spearman’s rho). The correlation coefficients strength (effect size) was considered poor if the Spearman’s rho was < 0.20, acceptable if 0.20–0.49 and good/strong if ≥0.50 in according to previously used dietary methods [29].

The agreement between methods was analyzed using Bland-Altman plots [30], using a plot of the mean difference of iodine intake between the two methods against the mean iodine intake of the two methods, also showing 95% limits of agreement (LOA). This was conducted to graphically assess the presence of bias or disagreement.

Agreement of quartile membership was assessed between estimated iodine intake from the I-FFQ and the food diary, estimated iodine intake from the I-FFQ and UIC, and estimated iodine intake from the food diary and UIC, using Cohen’s weighted kappa (k_w_), which takes the squared concordance of position among groups into account [31]. The weighted kappa was calculated using crosstab analysis and the script “Weighted Kappa, Kappa for ordered categories” available from the IBM website [32]. Stability between methods are presented as numbers and percentage of participants remaining in their quartile (stable quartile), in adjacent quartile, or in opposite quartile (two or more quartiles between, e.g. from first, to third or fourth quartile), compared to the other method selected. The criteria’s from Landis and Koch [33] were used to assess agreement, where a k_w_ of 0.01–0.20 represents slight agreement, 0.21–0.40 fair agreement, 0.41–0.60 moderate agreement, 0.61–0.80 substantial agreement, and 0.81–1.00 almost perfect agreement.

Linear regression analyses were used to assess the relationship between estimated iodine intake (including supplements) from the I-FFQ and thyroid markers: TSH, fT3 and fT4. Simple and adjusted (adjusted for BMI, age, nicotine use, and education) analyses are presented. Residual plots were examined and standardized residuals ±3 were excluded from the model (1 excluded from TSH, 2 excluded from fT4).

To assess the reproducibility of the I-FFQ we compared the estimated iodine intake from the I-FFQ completed at gestational week 18–19 with the I-FFQ completed at gestational week 35–36. The correlation of iodine intake from the I-FFQ were evaluated between the two time points, in addition to Bland-Altman plots. 

## Results

Characteristics of the pregnant women (gestational week 19) enrolled in the study are shown in Table 1.

**Table 1:** Baseline characteristics of pregnant women enrolled in Mommy’s Food

Characteristic	N	
Age (years), mean (SD)	135	29.3 (3.4)
Gestational weeks, mean (SD)	127	19.0 (1.3)
Pre-pregnancy BMI (kg/m^2^), median (p25-p75)	132	22.2 (20.6–24.3)
Marital status, n (%)	133	
Married		43 (32)
Cohabiting		85 (64)
Other		5 (4)
Education level, n (%)	133	
Elementary school		2 (1.5)
High school		17 (13)
≤ 4 years university/college		33 (25)
> 4 years university/college		81 (61)
Nicotine use in pregnancy ^a^, yes, n (%)	132	
≤ gestational week 8		12 (9)
> gestational week 8		0

^a^No participants reported use of nicotine after gestational week 8. BMI, body mass index; SD, standard deviation

Estimated iodine intake from the I-FFQ and food diary, and UIC and estimated iodine intake from UIC in the pregnant women are shown in Table 2. Median estimated iodine from the I-FFQ was 202 μg/day, which was significantly higher compared to estimated iodine intake from the food diary (151 μg/day) (*P* = 0.002). This was also higher compared to the estimated iodine intake from UIC (median 147 μg/day) (*P* = 0.001). The median estimated iodine intake from UIC (147 μg/day) was similar to the median total estimated iodine intake from the iodine specific food diary (151 μg/day) (same time period as urine spot samples) (*P* = 0.882).

**Table 2:** Descriptive of estimated iodine intake from the I-FFQ and food diary (μg/day), and urinary iodine concentration (UIC, μg/L) and estimated iodine intake from UIC (μg/day) in pregnant women (gestational week 18-19) enrolled in the Mommy’s Food study

	N	Mean (SD) ^f^	Median (p25-p75)	p5-p95
Estimated iodine intake, I-FFQ, μg/day ^a^				
Diet	124	134 (73)	123 (89–157)	51, 309
Diet and supplements	124	202 (108)	202 (106–275)	60, 377
Estimated iodine intake, food diary, μg/day ^b^				
Diet	134	116 (51)	105 (80–153)	42, 203
Diet and supplements	134	171 (99)	151 (87–262)	47, 342
Urinary iodine concentration (UIC), μg/L ^c^				
All participants	134	103 (56)	94 (62–130)	36, 210
Non-supplement users ^d^	87	89 (49)	77 (58–120)	31, 172
Supplements users ^d^	47	129 (58)	130 (77–160)	49–270
Estimated iodine intake from UIC, μg/day ^e^				
All participants	117	166 (93)	147 (104–206)	61, 404
Non-supplement users ^d^	75	135 (69)	119 (83–169)	46, 273
Supplement users ^d^	42	220 (105)	202 (143–262)	73, 439

^a^ Iodine specific food frequency questionnaire (I-FFQ)

^b^ Iodine specific food diary from six consecutive days

^c^ Pooled sample of spot urinary samples from six consecutive days

^d^ Reported use of iodine containing supplement in the food diary

^e^ Estimated from the equation: Urinary iodine concentration (μg/L) × 0.0235 × body weight (kg) (IOM 2001) [28]. Self-reported current body weight used for estimation (data of n = 17 missing)

^f^ Differences between the different methods were tested by Wilcoxon’s signed-rank test. Difference between estimated iodine intake from I-FFQ and food diary (without supplements): *P* = 0.030; estimated iodine intake from I-FFQ and food diary (with supplements): *P* = 0.002; estimated iodine intake from UIC and food diary: *P* = 0.882; estimated iodine intake from UIC and I-FFQ: *P* = 0.001

Correlations coefficients between estimated iodine intake from the I-FFQ, the food diary and UIC are presented in Table 3. There was a significant strong correlation between estimated iodine intake (including diet and supplements) from the I-FFQ and the food diary (*r* = 0.62, *P* < 0.001). Further, there was a significant acceptable correlation between estimated iodine intake from I-FFQ and UIC (*r* = 0.21, *P* = 0.018) and a significant acceptable correlation between estimated iodine intake from the food diary and UIC (*r* = 0.41, *P* < 0.001).

**Table 3:** Spearman’s rho correlation coefficient ^a^ between estimated iodine intake from I-FFQ (μg/day), estimated iodine intake from food diary (μg/day) and urinary iodine concentration (UIC) (μg/L)

Iodine intake from	I-FFQ^b^ vs. Food diary^c^ (n = 123)	I-FFQ^b^ vs. UIC^d^ (n = 123)	Food diary^c^ vs. UIC^d^ (n = 134)
Diet	0.36 (*P* < 0.001)	0.06 (*P* = 0.488)	0.18 (*P* = 0.042)
Diet and supplements	0.62 (*P* < 0.001)	0.21 (*P* = 0.018)	0.41 (*P* < 0.001)

^a^Spearman’s rank order correlation coefficient. The correlation coefficients strength (effect size) was considered poor if < 0.20, acceptable if 0.20–0.49 and strong if ≥0.50 in according to previously used dietary methods [30]

^b^Iodine specific food frequency questionnaire (I-FFQ)

^c^Iodine specific food diary from six consecutive days

^d^ Urinary iodine concentration (UIC): Pooled sample of spot urinary samples from six consecutive days

Table 4 presents the stability of quartile membership between the different methods. The stability was highest between the estimated iodine intake from I-FFQ and the food diary, where respectively 89% were classified into the same (47%) or adjacent (42%) quartile, showing a moderate to substantial agreement (k_w_ = 0.60). A fair agreement was seen between the I-FFQ and UIC (k_w_ = 0.21), and between the food diary and UIC (k_w_ = 0.40).

**Table 4:** Agreement of quartile membership between estimated iodine intake from the iodine-specific food frequency questionnaire (I-FFQ) and the food diary, and urinary iodine concentration (UIC)

	I-FFQ^a^ vs. Food diary^b^ (n = 122)	I-FFQ^a^ vs. UIC^c^ (n = 123)	Food diary^b^ vs. UIC^c^ (n = 133)
Stable quartile, *n* (%)	57 (47%)	41 (33%)	53 (40%)
Adjacent quartile, *n* (%)	51 (42%)	51 (42%)	50 (38%)
Opposite quartile, *n* (%)	14 (11%)	31 (25%)	30 (23%)
Weighted kappa, k_w_^d^	0.60	0.21	0.40

^a^Iodine specific food frequency questionnaire (I-FFQ)

^b^Iodine specific food diary from six consecutive days

^c^Urinary iodine concentration (UIC): Pooled sample of spot urinary samples from six consecutive days

^d^Tracking coefficient of Cohen’s weighted kappa. A k_w_ of 0.01–0.20 represents slight agreement, 0.21–0.40 fair agreement, 0.41–0.60 moderate agreement, 0.61–0.80 substantial agreement, and 0.81–1.00 almost perfect agreement [33]

**Fig. 1:**
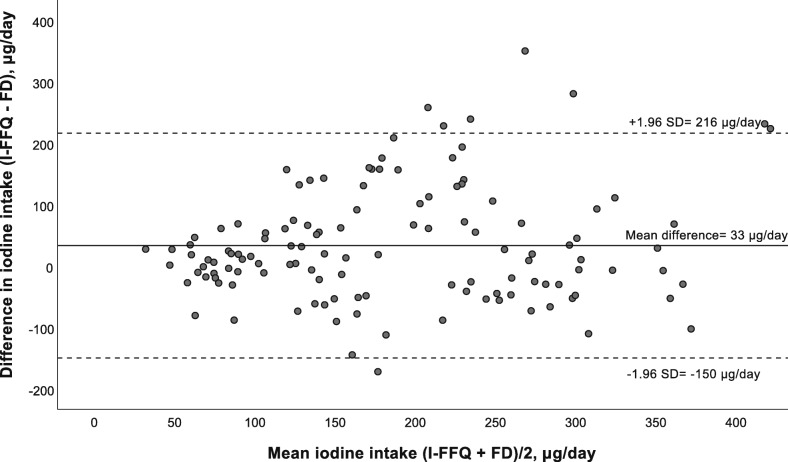
Bland-Altman plot of agreement between the iodine intake estimated from the iodine-specific food frequency questionnaire (I-FFQ) and the food diary (FD) (including diet and supplements) (n = 123). The solid line represents the mean difference between the two methods (33 μg/day), and the dotted lines represents the limits of agreements (LOA) corresponding to ±1.96 SD (lower agreement: − 150 μg/day, upper agreement: 216 μg/day)

The Bland-Altman plot comparing estimated iodine intake from I-FFQ and food diary (including diet and supplements) is presented in Fig. 1. The mean absolute difference (bias) in iodine intake between the methods was observed to 33 μg/day, with a LOA ranging from − 150 (lower LOA) to 216 (upper LOA) μg/day. The number of individuals observed to be beyond the LOA was 8 of 123, which corresponds to 6.5%.

**Fig. 2:**
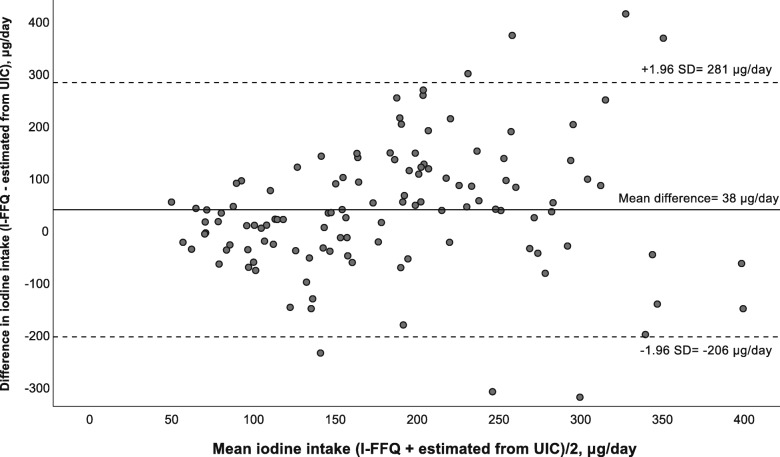
Bland-Altman plot of agreement between the iodine intake estimated the iodine-specific food frequency questionnaire (I-FFQ) and iodine intake estimated from UIC (n = 123). The solid line represents the mean difference between the two methods (38 μg/day), and the dotted lines represents the limits of agreements (LOA) corresponding to ±1.96 SD (lower agreement: − 206 μg/day, upper agreement: 281 μg/day)

The Bland-Altman plot comparing iodine intake estimated from the I-FFQ (including diet and supplements) and estimated iodine intake from UIC is presented in Fig. 2. The mean absolute difference (bias) in iodine intake between the methods was observed to 38 μg/day, with a LOA ranging from − 206 (lower LOA) to 281 (upper LOA) μg/day. The number of individuals observed to be beyond the LOA was 7 of 123, which corresponds to 5.7%.

**Fig. 3:**
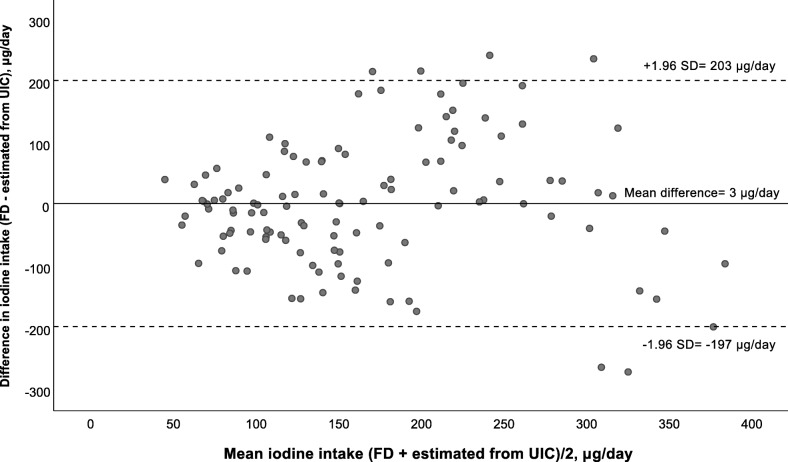
Bland-Altman plot of agreement between the iodine intake estimated from the food diary (FD) and iodine intake estimated from UIC (n = 134). The solid line represents the mean difference between the two methods (3 μg/day), and the dotted lines represents the limits of agreements (LOA) corresponding to ±1.96 SD (lower agreement: − 197 μg/day, upper agreement: 203 μg/day)

The Bland-Altman plot comparing iodine intake estimated from the food diary (including diet and supplements) and estimated iodine intake from UIC is presented in Fig. 3. The mean absolute difference (bias) in iodine intake between the methods was observed to 3 μg/day, with a LOA ranging from − 197 (lower LOA) to 203 (upper LOA) μg/day. The number of individuals observed to be beyond the (LOA) was 7 of 134, which corresponds to 5.2%.

Estimated iodine intake from the I-FFQ was positively associated with TSH, and negatively associated with fT3 and fT4, however neither of the associations were statistically significant after adjustments (Table 5).

**Table 5:** Association between estimated iodine intake from the I-FFQ and thyroid biomarkers^a^

	Unadjusted coefficients (95% CI)			Adjusted coefficients (95% CI)^b^		
		*P*	R^2^		*P*	R^2^
TSH (mIU/L)	22.2 (−3.2, 47.6)	*0.087*	0.024	12.5 (−12.0, 37.1)	*0.312*	0.107
fT3 (pmol/L)	−11.5 (− 54.2, 31.2)	*0.595*	0.002	−11.6 (−51.7, 28.5)	*0.567*	0.101
fT4 (pmol/L)	−5.0 (−16.2, 6.3)	*0.382*	0.006	−5.6 (−16.5, 5.3)	*0.314*	0.107

fT3, free triiodothyronine; fT4, free thyroxine, TSH, thyroid stimulating hormone

^a^n = 123 for TSH; n = 124 for fT3; n = 122 for fT4

^b^Adjusted for BMI, age, nicotine use while pregnant and education. Categories for nicotine use: 0 = no, 1 = yes; categories for education 0 ≤ high school, 1 ≥ university/ university college

There was no difference between the estimated iodine intake from the I-FFQ at gestational week 18–19 and gestational week 35–36 (median (p25-p75) iodine intake: 145 (90–267) vs. 152 (115–258), *P* = 0.866, n = 47). In addition, there was a strong correlation between estimated iodine intake from the I-FFQ between these two time points (*r* = 0.63, *P* < 0.001, n = 47). The mean absolute difference (bias) in iodine intake between the methods was observed to − 5 μg/day, with a LOA ranging from − 177 (lower limit of agreement) to 167 (upper LOA) μg/day. The number of individuals observed to be beyond the LOA was 5 of 47 (5.2%).

## Discussion

This study suggests that the I-FFQ can be used as a valid tool to estimate and rank iodine intake among Norwegian pregnant women, because of its association with estimated iodine intake from a 6-days structured food diary and UIC from six spot urine samples. The I-FFQ further showed strong reproducibility. To the best of our knowledge, there is only one previously validated I-FFQ for pregnant women, which was developed in Australia [34]. A comprehensive FFQ has previously been used and validated for iodine intake in the Norwegian Mother and Child Cohort study [21, 22], however, this is a full FFQ consisting of 255 food items [22]. Therefore, this I-FFQ could be a useful tool, as it presents a lower burden for the participants. There are few validated tools available to asses iodine intake in an efficient manner. UIC is the most common method for assessing iodine status [1]. However, an I-FFQ has the advantage to measure long-term dietary intake in a simple, cost- and time-efficient manner in a large sample size that is geographically widespread [16]. As iodine is present in few foods, and iodized salt is used in negligible amounts in Norway, dietary assessment tools may be a promising method to evaluate iodine intake through dietary reference intakes in a population. An I-FFQ along with the use of UIC in a population, can also give a better understanding of current iodine status and iodine sources in population studies [23].

Weighed food- or diet records are considered the gold standard in dietary research, and is the first methods of choice for validation of FFQ data [18]. According to Gibson, a 7-days weighted food record is considered the best reference method [35]. We only used a 6-days structured food diary including 28 food items of iodine rich food groups as the dietary reference method. Though, in most settings, using more than four to five observations (days) of the reference methods per subject has also been considered sufficient [36]. The agreement between the I-FFQ and the food diary was moderate to substantial (*k* = 0.60). When assessing validation of specific nutrients from an FFQ, a weighted kappa value above 0.4 has been considered as acceptable [29, 37]. The kappa value was in accordance with the correlation coefficients as there was a strong correlation between estimated total iodine intake from the I-FFQ and food diary (*r* = 0.62, *P* < 0.001). This was mostly higher or comparable to what is reported in other studies comparing iodine intake from an FFQ against a food diary or food record (correlation coefficients ranging from 0.26–0.88) [21, 34, 38–41].

The mean absolute difference between the methods showed that the estimated iodine intake was highser from the I-FFQ, compared to the food diary and estimated iodine intake from UIC. A higher iodine intake estimated from an FFQ compared to other dietary reference methods has also been found in other studies [39, 42, 43], while in some studies FFQ have not been found to overestimate [22, 34, 40]. There could be several reasons of why the I-FFQ estimated a higher iodine intake compared to the food diary. The records of the I-FFQ and the food diary was from different time periods (approximately four months vs. six days) and the I-FFQ was completed retrospective while the food diary was completed day-by-day. In addition, the I-FFQ assessed long-term habitual iodine intake, included more food items and covered rarely consumed food items such as several seafood species and items (e.g. reporting of an intake 1–3 times/months) which the food diary may not has covered. The time period of pregnancy have also been shown to be a period where there is a potential for dietary changes [44–47]. Thus, we cannot conclude whether the estimated higher iodine intake from the I-FFQ compared to the food diary was caused by an overestimation from the I-FFQ, reporting of rarely consumed food items which the food diary did not cover, or if it was an actual difference in intake between the two time periods. It should also be noted that we did not adjust for energy intake when calculating iodine intake, as we did not complete a full FFQ or food diary, which further is a limitation in our study. In both the I-FFQ and food diary, we only included iodine intake from milk and dairy products, seafood, eggs and supplements. These are the most important food groups of iodine intake in Norway, however other food groups (such as e.g. bread, cereals, snack, vegetables) may also contribute with some iodine [10], and the exclusion of these food groups may on the other hand contributed to an underestimation of iodine intake.

There was a significantly higher estimated iodine intake from the I-FFQ and food diary when supplements was added to the summarization. In addition, the correlations coefficients were strengthened. This is also found in other studies [21, 34] and highlights the importance of including supplements in dietary research studies of iodine as it contributes with a significant amount of intake.

Correlation between methods only assess the degree of the association between them, and not the agreement between them. In contrast, the Bland-Altman plot is a preferred method to assess agreement between two dietary reference methods across the range of intakes [18]. From the Bland-Altman plots (Figs. 1, 2 and 3), the LOA were large and for all plots there seemed to be a systematic increase in the difference between the two methods with increasing iodine intake. This may indicate that estimating iodine intake is more difficult when intake is higher. This may also be explained by the fact that when several sources of intake are included, the accuracy of the dietary intake estimations decrease as an increased number of potential errors are introduced, e.g. inaccurate portion estimations or food composition data. This have also been confirmed by other studies validating FFQs [38, 39]. However, few participants (5.3–6.5%) fell outside the LOA (±1.96 SD) which is indicated as the accepted level of agreement [30, 48].

A biomarker can provide an estimate of dietary intake that is objective and independent of the subject’s reported dietary intake [18, 49]. We found a fair agreement between UIC and iodine intake from the I-FFQ (*k* = 0.205) and the food diary (*k* = 0.399). In addition, there was an acceptable correlation between UIC and iodine intake from the I-FFQ (*r* = 0.213, *P* = 0.018) and the food diary (*r* = 0.408, *P* < 0.001). The use of spot urine samples to measure UIC as a marker of iodine status has its limitations owing to large inter- and intra-individual variations. A strength to this study is that the participants collected six spot urinary samples on consecutive days, and not a single spot urine sample. However, it has been suggested that at least ten spot urine samples or 24-h urine collections, is needed to account for intra- and inter individual variability [50]. Nonetheless, this is demanding for the participants and was not prioritized in this study. Consequently, we cannot exclude that the UIC has limitations regarding within day, and day-to-day variation. The iodine intake from the food diary had the highest relative validity, as it showed the highest correlation and agreement with the UIC. This was also expected as the sampling of spot urine samples was collected of the same days as the food diary was conducted (six consecutive days).

We also assessed the association between thyroid function tests (TSH, fT3, fT4) with estimated iodine intake from the I-FFQ. The direction of the associations were similar to what was reported in another study of Norwegian pregnant women, where an inverse association was seen between UIC and fT3, and fT4, and a positive association between UIC and TSH [51]. However, we did not find any significant associations, similarly as reported by others [34, 51]. TSH and thyroid hormones are not considered sensitive markers of iodine status and may only be affected if severe iodine deficiency is present [1]. Thus, a significant association was not be expected.

To determine whether an FFQ shows reproducible results, reproducibility should always be assessed [18]. There was a strong association between estimated iodine intake from the I-FFQ at gestational week 18–19 with gestational week 35–36. Further, no significant differences in estimated iodine intake from the I-FFQ between the two time points were found. However, as this paper was part of an RCT we were only able to assess reproducibility in the control group of the study (*n* = 47). Thus, the reduced number of participants assessing the reproducibility of the I-FFQ is a limitation.

The strengths in this study are the use of both a dietary method (food diary) and biomarkers (UIC and thyroid function tests) to validate the I-FFQ, as using more than one approach to validate an FFQ gives further credibility to the results [18]. We also used multiple statistical tests, which is considered a strength when evaluating validity of dietary methods [29]. In addition, we used up to date chemical analyses of most food items to estimate iodine intake [15]. We included portion sizes in the I-FFQ which is recommended when calculating nutrient intake [18]. Further, the sample size was considered adequate for validation of an FFQ [18]. However, the study group may not be a representative sample of pregnant women in Norway as this was a group with high socioeconomic status. Still, the study group has a broad range of iodine intakes (Table 2), and we believe that this I-FFQ may also be valid in other populations with similarly dietary patterns where salt is not iodized, such as the general adult population in Norway.

## Conclusion

In summary, this study suggests that the I-FFQ can be used as a valid tool to estimate and rank iodine intake among Norwegian pregnant women, because of its acceptable correlation and agreement with estimated iodine intake from a 6-days structured food diary and UIC. The I-FFQ further showed strong reproducibility. Thus, this I-FFQ can be used as a tool to evaluate and monitor iodine intake in this population. As iodine deficiency is re-emerging in Norway and Europe, there is a need for validated tools that are non-invasive, simple, cost- and time-efficient to evaluate iodine intake and to identify women at risk of inadequate intake.

## Supplementary information

**Additional file 1: Figure S1.** Overview of methods used in validation of the I-FFQ in Norwegian pregnant women. **Table S1.** Number of food items specified in the I-FFQ and the food diary, and number of frequency alternatives in the I-FFQ.

**Additional file 2: Table S2.** Iodine content in food items used for calculation of iodine intake from the I-FFQ. **Table S3.** Iodine content in food items used for calculation of iodine intake from the food diary.

## Data Availability

The datasets used and analysed during the current study are available from the corresponding author on reasonable request.

## Abbreviations

BMI: Body mass index
FFQ: Food frequency questionnaire
fT3: Free triiodothyronine
fT4: Free thyroxine
ICP-MS: Inductively coupled plasma mass spectrometry
I-FFQ: Iodine-specific food frequency questionnaire
IMR: Institute of Marine Research;
RCT: Randomized controlled trial
SD: Standard deviation
TSH: Thyroid stimulating hormone
UIC: Urinary iodine concentration
WHO: World Health Organization
