# Supplementary material for: Validation and reproducibility of a new iodine specific food frequency questionnaire for assessing iodine intake in Norwegian pregnant women
Source: Nutr J. 2019 Oct 29;18:62. doi: 10.1186/s12937-019-0489-4 (PMC6821006; doi:10.1186/s12937-019-0489-4)
Supplement: Supplementary file 1 — Additional file 1: Figure S1. Overview of methods used in validation of the I-FFQ in Norwegian pregnant women. Table S1. Number of food items specified in the I-FFQ and the food diary, and number of frequency alternatives in the I-FFQ. [file 12937_2019_489_MOESM1_ESM.docx]

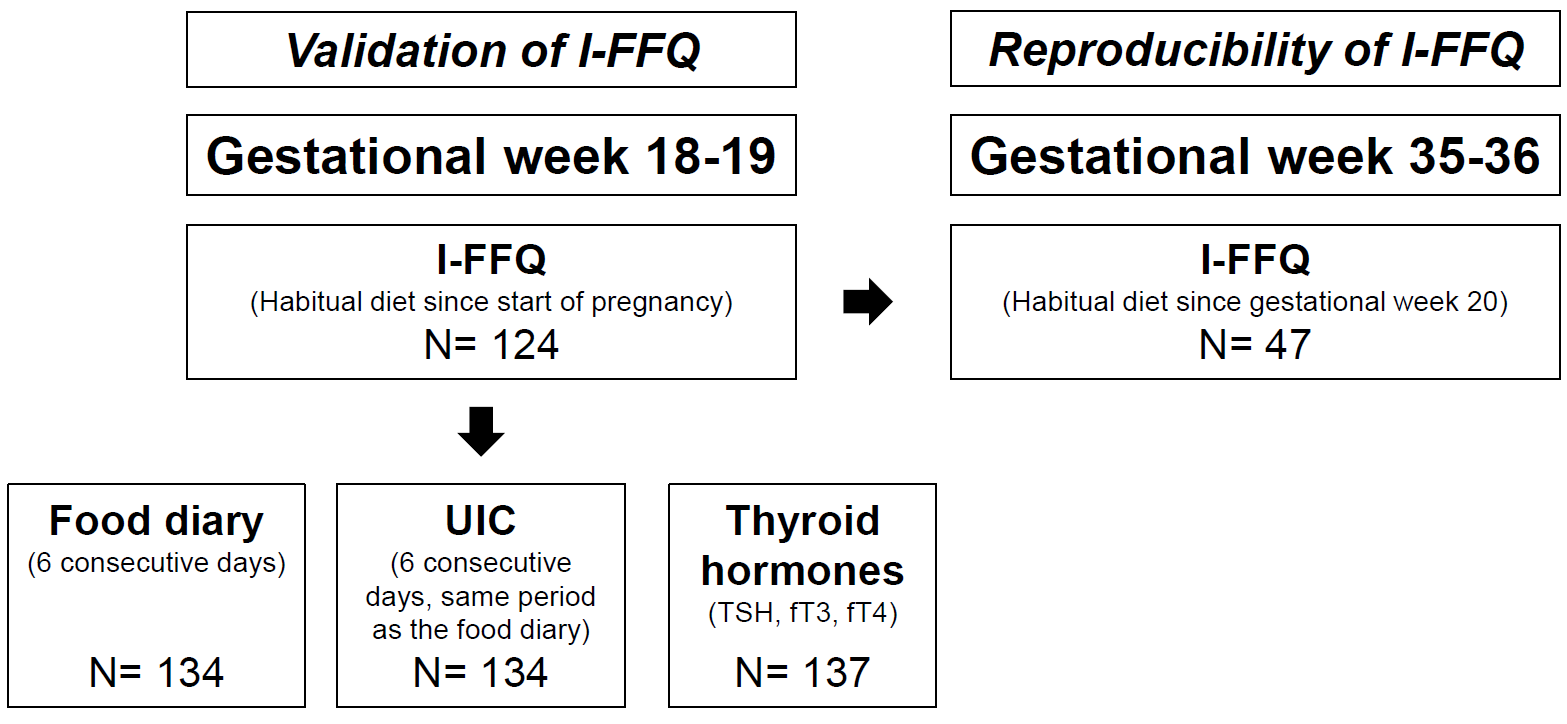


**Figure S1** Overview of methods used in validation of the I-FFQ in Norwegian pregnant women

I-FFQ, iodine specific food frequency questionnaire; TSH, thyroid stimulating hormone; fT3, free triiodothyronine; fT4, free thyroxine

**Table S1** Number of food items specified in the iodine specific food frequency questionnaire (I-FFQ) and the food diary, and number of frequency alternatives in the I-FFQ.

| **Food item** | **Food diary - Number of food items** | **I-FFQ - Number of food items** | **Frequency response in I-FFQ** |
| --- | --- | --- | --- |
|  |  |  |  |
| Seafood as dinner or warm lunch | 5 | 21 | 5 frequency alternatives:   - Never - Less than 1/month - 1-3 times/month - 1-2 times/week - 3 times or more/week |
| Seafood as spread | 4 | 14 | 5 frequency alternatives:   - Never - Less than 1/month - 1-3 times/month - 1-2 times/week - 3 times or more/week |
| Milk, yoghurt and other milk containing drinks | 6 | 13 | 7 frequency alternatives:   - Never - Less than 1/week - 1-3 times/week - 4-6 times/week - 1 time/day - 2 times/day - 3-4 times or more/day |
| Cheese and cheese products | 8 | 7 | 7 frequency alternatives:   - Never - Less than 1/week - 1-3 times/week - 4-6 times/week - 1 time/day - 2 times/day - 3-4 times or more/day |
| Other foods made with milk and dairy products | 5 | 4 | 7 frequency alternatives:   - Never - Less than 1/week - 1-3 times/week - 4-6 times/week - 1 time/day - 2 times/day - 3-4 times or more/day |
| Eggs | 1 | 1 | 6 frequency alternatives:   - Less than 1 egg/week - 2-3 eggs/week - 4-5 eggs/week - 6-7 eggs/week - 8 or more eggs/week |
| **Total food items** | 28 | 60 |  |

In addition, the I-FFQ and the food diary included questions regarding dietary supplements including type, brand and intake frequency.
